# Brain region and gene dosage-differential transcriptomic changes in *Shank2*-mutant mice

**DOI:** 10.3389/fnmol.2022.977305

**Published:** 2022-10-13

**Authors:** Ye-Eun Yoo, Taesun Yoo, Hyojin Kang, Eunjoon Kim

**Affiliations:** ^1^Center for Synaptic Brain Dysfunctions, Institute for Basic Science (IBS), Daejeon, South Korea; ^2^Division of National Supercomputing, Korea Institute of Science and Technology Information (KISTI), Daejeon, South Korea; ^3^Department of Biological Sciences, Korea Advanced Institute of Science and Technology (KAIST), Daejeon, South Korea

**Keywords:** autism spectrum disorder, *Shank2*, cortex, hippocampus, striatum, gene dosage, transcriptome, RNA-seq

## Abstract

Shank2 is an abundant excitatory postsynaptic scaffolding protein that has been implicated in various neurodevelopmental and psychiatric disorders, including autism spectrum disorder (ASD), intellectual disability, attention-deficit/hyperactivity disorder, and schizophrenia. *Shank2*-mutant mice show ASD-like behavioral deficits and altered synaptic and neuronal functions, but little is known about how different brain regions and gene dosages affect the transcriptomic phenotypes of these mice. Here, we performed RNA-Seq-based transcriptomic analyses of the prefrontal cortex, hippocampus, and striatum in adult *Shank2* heterozygous (HT)- and homozygous (HM)-mutant mice lacking exons 6–7. The prefrontal cortical, hippocampal, and striatal regions showed distinct transcriptomic patterns associated with synapse, ribosome, mitochondria, spliceosome, and extracellular matrix (ECM). The three brain regions were also distinct in the expression of ASD-related and ASD-risk genes. These differential patterns were stronger in the prefrontal cortex where the HT transcriptome displayed increased synaptic gene expression and reverse-ASD patterns whereas the HM transcriptome showed decreased synaptic gene expression and ASD-like patterns. These results suggest brain region- and gene dosage-differential transcriptomic changes in *Shank2*-mutant mice.

## Introduction

Transcriptomic analyses of brain samples from individuals with autism spectrum disorder (ASD) have provided substantial insights into the convergent biological functions and pathways that are dysregulated in ASD, which include chromatin regulation, transcription, translation, synapse function, neuronal and glial development, and immune function (Garbett et al., 2008; Voineagu et al., 2011; Parikshak et al., 2013, 2016; Gupta et al., 2014; Krishnan et al., 2016; Velmeshev et al., 2019). Studies on mouse models of ASD have also revealed altered transcriptomic patterns that are associated with dysregulated biological functions and pathways (Jiang and Ehlers, 2013; Barnard et al., 2015; Bourgeron, 2015; Lee et al., 2015a,^2017^; Nelson and Valakh, 2015; Sahin and Sur, 2015; Hulbert and Jiang, 2016; Monteiro and Feng, 2017; Varghese et al., 2017; Mossa et al., 2018; Winden et al., 2018; Bagni and Zukin, 2019; Basilico et al., 2020; Rein and Yan, 2020; Takumi et al., 2020; Moffat et al., 2021; Yan and Rein, 2021) However, relatively little is known about how controllable parameters in mouse models of ASD, such as age, brain region, and dosage, can affect brain transcriptomic patterns. Such knowledge would be expected to facilitate the appropriate design and data interpretation of future experiments.

Shank2, also known as ProSAP1, is an abundant scaffolding protein that is enriched in the postsynaptic density of an excitatory synapse (Du et al., 1998; Boeckers et al., 1999; Lim et al., 1999; Naisbitt et al., 1999; reviewed in Sheng and Kim, 2000, 2011; Boeckers et al., 2002; Grabrucker et al., 2011; Sala et al., 2015; Mossa et al., 2017; Eltokhi et al., 2018). Shank2 has been implicated in various brain disorders, including ASD, intellectual disability, developmental delay, attention deficit/hyperactivity disorder, schizophrenia, epilepsy, and obsessive compulsive disorder (Berkel et al., 2010, 2011; Pinto et al., 2010; Leblond et al., 2012, 2014; Rauch et al., 2012; Sanders et al., 2012; Chilian et al., 2013; Liu et al., 2013; Guilmatre et al., 2014; Costas, 2015; Peykov et al., 2015a,b; Homann et al., 2016; Bowling et al., 2017; Mossa et al., 2017; Yuen et al., 2017; Bai et al., 2018; Guo et al., 2018; Lu et al., 2018; Callaghan et al., 2019; Satterstrom et al., 2020; Trost et al., 2020; Wang et al., 2020; Ma et al., 2021; Ohashi et al., 2021; Chenbhanich and So, 2022). Previous studies in mouse/rat models of ASD and human neurons deficient of Shank2 have suggested various molecular, synaptic, neuronal, and circuit mechanisms underlying Shank2-related phenotypes (Schmeisser et al., 2012; Won et al., 2012; Ey et al., 2013, 2018; Lee et al., 2015b,^2018^, ^2020^, 2021a,b; Ferhat et al., 2016; Ha et al., 2016; Ko et al., 2016; Peter et al., 2016; Lim et al., 2017; Pappas et al., 2017; Sato et al., 2017, 2020; Yoon et al., 2017; Heise et al., 2018; Kim et al., 2018; Modi et al., 2018; Wegener et al., 2018; Chung et al., 2019; de Chaumont et al., 2019; Zaslavsky et al., 2019; Chen et al., 2020; Han et al., 2020; Daini et al., 2021; Grabrucker et al., 2021; Horner et al., 2021; Lutz et al., 2021; Yoo et al., 2021; Wahl et al., 2022) [reviewed in (Boeckers et al., 2002; Grabrucker et al., 2011; Jiang and Ehlers, 2013; Guilmatre et al., 2014; Yoo et al., 2014; Sala et al., 2015; Schmeisser, 2015; Monteiro and Feng, 2017; Mossa et al., 2017; Eltokhi et al., 2018; Ey et al., 2020; Jung and Park, 2022)]. However, it remains unclear whether and how different brain regions distinctly contribute to the observed Shank2-related mouse phenotypes. In addition, heterozygous Shank2 deletion in mice has been shown to induce much weaker changes in behavioral phenotypes relative to a homozygous gene deletion (Schmeisser et al., 2012; Won et al., 2012; Pappas et al., 2017). However, because autistic human individuals usually carry heterozygous SHANK2 mutations, it is important to test if heterozygous Shank2 deletion in mice leads to detectable changes in molecular, neuronal, and/or synaptic phenotypes. In addition, we previously performed transcriptomic analyses of the medial prefrontal brain regions from heterozygous and homozygous Shank2-mutant mice lacking exons 6 and 7. We observed stronger reverse-ASD-like transcriptomic changes in heterozygous mutant mice than in homozygous mutant mice, which were more prominent at juvenile stages relative to adult stages (Lee et al., 2021b). This led us to question whether such changes are conserved across a larger prefrontal area and/or in other brain regions.

In the present study, we set out to perform transcriptomic analyses of the prefrontal cortex (termed cortex hereafter), hippocampus, and striatum regions of adult (∼postnatal day 90 or P90) *Shank2* HT- and HM-mutant mice lacking exons 6–7. Our findings collectively indicate brain region and gene dosage-differential transcriptomic patterns in *Shank2*-mutant mice.

## Materials and methods

### Animals

*Shank2*-mutant mice lacking exons 6–7 have been reported previously (Won et al., 2012) and have been deposited at the Jackson Laboratory (B6N.129S4-*Shank2*^*TM*1*Mgle*^/CsbdJ; Jackson 033667). Mice were maintained at the mouse facility of the Korea Advanced Institute of Science and Technology (KAIST); they were fed *ad libitum* and maintained according to the Animal Research Requirements of KAIST.

### RNA-seq analysis

Transcript abundance was estimated with Salmon (v1.1.0) (Patro et al., 2017) in Quasi-mapping-based mode onto the *Mus musculus* (mouse) genome (GRCm38) with GC bias correction (–gcBias). Quantified gene-level abundance data was imported to R (v.4.1.3) with the tximport (Soneson et al., 2015) package and differential gene expression analysis was carried out using R/Bioconductor DEseq2 (v1.30.1) (Love et al., 2014). Normalized read counts were computed by dividing the raw read counts by size factors and fitted to a negative binomial distribution. The *P*-values were adjusted for multiple testing with the Benjamini–Hochberg correction. Genes with an adjusted *P*-value of less than 0.05 were considered as differentially expressed. Volcano plots were generated using the R ggplot2 (v.3.3.3) package. The gene ontology (GO) enrichment analyses were performed using database for annotation, visualization, and integrated discovery (DAVID) (version 6.8) (Huang et al., 2009) using *Mus musculus* (mouse) as a background.^1^

Synaptic gene ontologies (SynGO) analysis^2^ was performed to associate synapse functions to the identified DEGs. Comparison to all brain-expressed genes was used to determine the extent of enrichments for SynGO terms. The gene count indicates the number of unique genes from the input gene list against SynGO, or child, terms and was used to build the sunburst plot color-coded by gene counts. STRING analysis was used to obtain and visualize protein-protein interaction (PPI) plots for SynGO-DEGs.^3^

Gene set enrichment analysis (GSEA)^4^ (Subramanian et al., 2005) was used to determine whether priori-defined gene sets would show statistically significant differences in expression between wild-type (WT) and *Shank2*-mutant mice. Enrichment analysis was performed using GSEAPreranked (gsea-3.0.jar) module on gene set collections downloaded from the Molecular Signature Database (MSigDB) v7.4.^5^ GSEA Preranked was applied using the list of all genes expressed, ranked by the fold change, and multiplied by the inverse of the *P*-value with recommended default settings (1,000 permutations and a classic scoring scheme). The false discovery rate (FDR) was estimated to control the false positive finding of a given normalized enrichment score (NES) by comparing the tails of the observed and null distributions derived from 1,000 gene set permutations. The gene sets with an FDR of less than 0.05 were considered as significantly enriched. Integration and visualization of the GSEA results were performed using the EnrichmentMap Cytoscape App (version 3.8.1) (Merico et al., 2010; Isserlin et al., 2014).

## Results

### Differentially expressed genes (DEGs) in Shank2-HT and Shank2-HM transcripts

**FIGURE 1:**
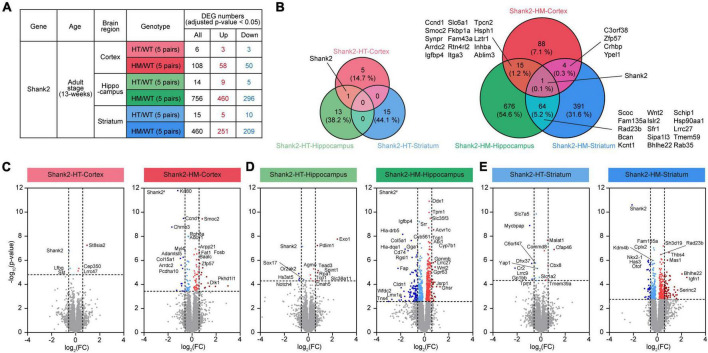
Differentially expressed genes (DEGs) among Shank2-heterozygous (HT) and Shank2-homozygous (HM) transcripts in the cortex, hippocampus, and striatum. **(A)** Numbers of DEGs from cortical, hippocampal, and striatal regions in Shank2-HT and Shank2-HM mice relative to wild-type (WT) [13 weeks; male; *n* = 5 mice (WT), 5 (HT/heterozygote), and 5 (HM/homozygote)]. DEGs were defined as genes showing transcript changes above the cutoff (adjusted *p*-value < 0.05). **(B)** Venn diagrams showing the overlaps of DEGs between cortical, hippocampal, and striatal regions in Shank2-HT and Shank2-HM mice [13 weeks; male; *n* = 5 mice (WT), 5 (HT), and 5 (HM)]. **(C–E)** Volcano plot representations of DEGs from cortical, hippocampal, and striatal transcripts in Shank2-HT and Shank2-HM mice. The DEGs (*p*-value < 0.05) were further color-coded to indicate those with strong fold changes (>1.5). Note that *Shank2* was not indicated as a DEG in some volcano plots (HT-Striatum) because of the low adjusted *p*-values (*p* = 0.2850), although their *p*-values were below 0.05 (*p* = 0.0019). Shank2^#^ in HM-Cortex and HM-Hippocampus volcano plots indicate very small *p*-values [-log_10_ (adjusted *p*-value) = 26 and 28 for HM-Cortex and HM-Hippocampus, respectively].

To explore whether different brain regions distinctly contribute to the various phenotypes observed in *Shank2*-mutant mice, we performed RNA-seq analysis of the prefrontal cortex (termed cortex hereafter), hippocampus, and striatum in Shank2-HT and Shank2-HM mice lacking exons 6–7 (*n* = 5 mice) at an adult stage (∼postnatal day 90 or P90) (Figure 1A and Supplementary Table 1). These three groups of transcripts were well separated in a heatmap analysis (Supplementary Figure 1).

The RNA-seq results were first analyzed for genes that were differentially expressed (DEGs) between WT mice and Shank2-HT or Shank2-HM mice. In the cortex, the Shank2-HT and Shank2-HM transcripts contained a total of 6 and 108 DEGs (adjusted *p* < 0.05), respectively, relative to WT (Figure 1A and Supplementary Table 2). Hippocampal Shank2-HT and Shank2-HM transcripts contained 14 and 756 DEGs, respectively, while striatal Shank2-HT and Shank2-HM transcripts contained 15 and 460 DEGs, respectively (Figure 1A). These results suggest that HM *Shank2* deletion is associated with more DEGs in all three brain regions compared to HT *Shank2* deletion.

The overlaps of the DEGs between different brain regions were small, ranging from ∼6 to 20% between Shank2-HT/HM cortical, hippocampal, and striatal transcripts (Figure 1B). The small overlaps between different brain regions were also supported by small correlations of fold changes (FC) between two groups of transcripts (Supplementary Figure 2). The hippocampus and striatum tended to share more DEGs than did the hippocampus/striatum and the cortex. *Shank2* was the only DEG shared by all three brain regions.

Volcano plots of the DEGs further highlighted the stronger transcriptomic impacts of HM *Shank2* deletion relative to HT *Shank2* deletion (Figures 1C–E).

### Database for annotation, visualization, and integrated discovery (DAVID) analysis of Shank2-HT and Shank2-HM DEGs

**FIGURE 2:**
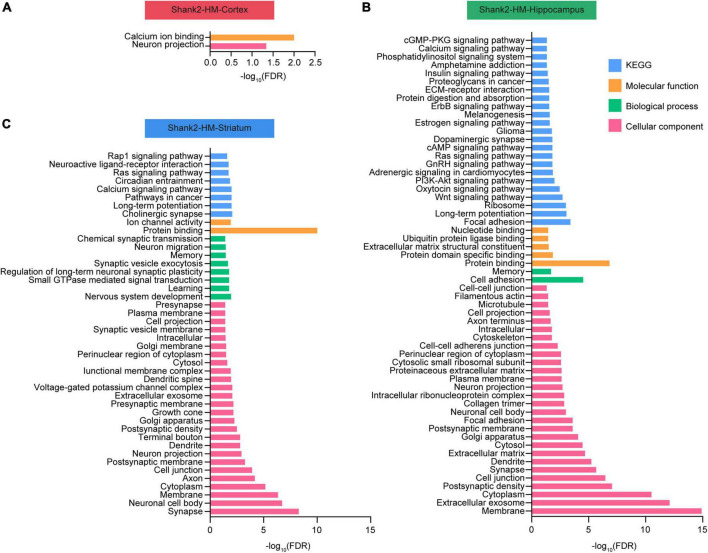
Database for annotation, visualization, and integrated discovery (DAVID) analysis of Shank2-heterozygous (HT) and Shank2-homozygous (HM) differentially expressed genes (DEGs). **(A–C)** DEGs in the cortical, hippocampal, and striatal transcripts from Shank2-HT and Shank2-HM mice [13 weeks; male; *n* = 5 mice wild-type (WT), 5 (HT), and 5 (HM)] were subjected to DAVID analysis for associations with gene ontology (GO) terms in the molecular function (MF), biological process (BP), cellular component, and KEGG domains. The low GO scores for the cortical DEGs may reflect the small number of these DEGs. FDR, false discovery rate.

Database for annotation, visualization, and integrated discovery analyses were performed using the Shank2-HM cortical, hippocampal, and striatal DEGs. Our DAVID analysis of the cortical Shank2-HM transcripts revealed only minimal enrichments in the GO terms (Figure 2A). In contrast, the hippocampal Shank2-HM transcripts were strongly enriched for GO terms associated with neuronal synapses, such as “postsynaptic density,” “synapse,” and “postsynaptic membrane,” as well as with subneuronal regions/structures, such as “dendrite,” “neuronal cell body,” and “neuronal projection” in the cellular component domain (Figure 2B). Another strong association was “protein binding” in the molecular function (MF) domain, suggesting that there may be alterations in multi-molecular synaptic interactions.

The results from the striatum indicated enhancement for GO terms associated with neuronal synapses, such as “synapse,” “postsynaptic membrane,” and “postsynaptic density,” along with subneuronal regions/structures, such as “neuronal cell body,” “axon,” and “dendrite” in the cellular component domain (Figure 2C). Another strong association was “protein binding” in the molecular function domain.

These results suggest that the Shank2-HM hippocampal and striatal DEGs are functionally similar to one another.

### Synaptic gene ontologies (SynGO) and protein-protein interaction (PPI) analyses of Shank2-HT and Shank2-HM DEGs

Given that our DAVID analyses of DEGs in Shank2-HM hippocampal/striatal transcripts yielded numerous synapse-associated GO terms, we further analyzed the Shank2-HT/HM DEGs using SynGO, which is an evidence-based and expertly curated resource for synaptic function and gene enrichment analyses (Koopmans et al., 2019).

Substantial proportions of the Shank2-HT/HM cortical/hippocampal/striatal DEGs belonged to SynGO genes (SynGO-DEGs), including 33/19% of Shank2-HT/HM cortical DEGs, 7/18% of Shank2-HT/HM hippocampal DEGs, and 7/19% of Shank2-HT/HM striatal DEGs (Supplementary Table 2). Notably, the enrichment levels of the differentially expressed SynGO genes were similar in the three brain regions for Shank2-HM (but not Shank2-HT) DEGs with sufficient numbers.

**FIGURE 3:**
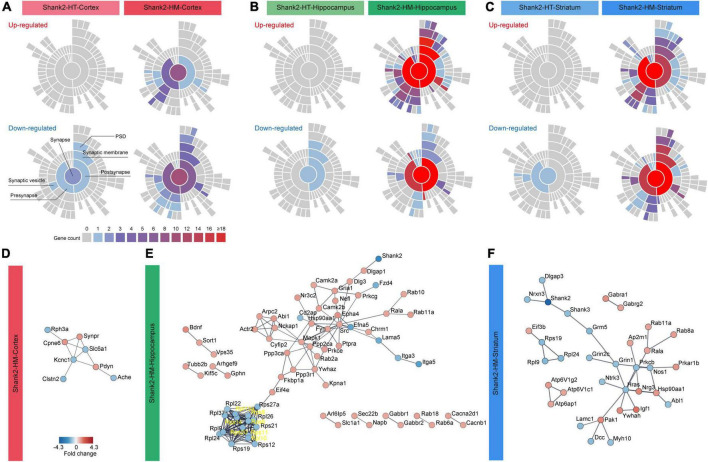
Synaptic gene ontologies (SynGO) and protein-protein interaction (PPI) analyses of Shank2-heterozygous (HT) and Shank2-homozygous (HM) differentially expressed genes (DEGs). **(A–C)** Sunburst plot visualization of the SynGO results, highlighting the extents of pre/postsynaptic functions in the up/downregulated DEGs belonging to SynGO genes (SynGO-DEGs) of cortical, hippocampal, and striatal regions of Shank2-HT and Shank2-HM mice [13 weeks; male; *n* = 5 mice wild-type (WT), 5 (HT), and 5 (HM)]. **(D–F)** PPI analysis of cortical, hippocampal, and striatal Shank2-HM SynGO-DEGs. Differential cutoff values were used for different brain regions to increase the visibility of the PPI clusters [medium confidence (0.400) for HM-Cortex and high confidence (0.900) for HM-hippocampus and HM-striatum]. Red and blue colors indicate up and down fold changes, respectively.

Functional analysis of SynGO-DEGs using sunburst plotting revealed distinct synaptic functions. These were more evident in large SynGO-DEG datasets, such as those for Shank2-HM SynGO-DEGs versus Shank2-HT SynGO-DEGs and hippocampal/striatal SynGO-DEGs versus cortical SynGO-DEGs (Figures 3A–C). Specifically, the upregulated hippocampal Shank2-HM SynGO-DEGs were enriched for pre- and postsynaptic functions, whereas the downregulated hippocampal Shank2-HM SynGO-DEGs were more strongly enriched for postsynaptic functions compared to presynaptic functions.

The upregulated striatal Shank2-HM DEGs were more strongly enriched for presynaptic functions. The downregulated striatal Shank2-HM SynGO-DEGs were more strongly enriched for postsynaptic functions, similar to the hippocampus. The up- and downregulated cortical Shank2-HM SynGO-DEGs were also distinctly enriched for pre- and postsynaptic functions, although this conclusion is less reliable given that the dataset was relatively small (20 genes in the cortex relative to 135 genes in the hippocampus and 89 genes in the striatum).

Protein–protein interaction analysis of the hippocampal and striatal SynGO-DEGs revealed distinct PPI patterns (Figures 3D–F). The hippocampal Shank2-HM SynGO-DEGs formed a cluster of postsynaptic scaffolding/receptor genes, including *Shank2*, *Dlgap1* (SAPAP1/GKAP), *Dlg3* (encoding SAP102), *Gria1* (GluA1 subunit of AMPA receptors), *Camk2a/b* (calcium/calmodulin-dependent protein kinase IIα/β), and *Shisa6* (encoding an AMPA receptor auxiliary protein) (Figure 3E). With the exception of *Shank2*, these genes tended to be upregulated. Another notable cluster that is linked to the abovementioned cluster contained many genes encoding signaling proteins such as *Src* (Src proto-oncogene, non-receptor tyrosine), *Fyn* (Fyn proto-oncogene, Src family tyrosine kinase), *Mapk1* (mitogen-activated protein kinase 1), *Ppp2ca* (protein phosphatase 2 catalytic subunit alpha) as well as actin-related genes (*Abi1*, *Cyfip2*, *Actr2*, and *Arpc2*), which are mostly upregulated. A downregulated cluster that is largely independent from the abovementioned clusters contained genes encoding ribosomal subunit proteins (*Rpl* and *Rps*).

Protein–protein interaction analysis of the striatal Shank2-HM SynGO-DEGs revealed a cluster of genes, including *Shank2*, *Shank3*, *Dlgap3* (SAPAP3), *Nrxn3* (Neurexin 3), *Grin1* (GluN1 subunit of NMDARs), and *Grm5* (metabotropic glutamate receptor 5), which are downregulated. Unlike our findings in the hippocampus, there was no independent cluster of *Rpl* or *Rps* genes.

The results of our SynGO and PPI analyses of hippocampal and striatal Shank2-HM DEGs collectively suggest that hippocampal and striatal Shank2-HM DEGs are: (1) strongly enriched for SynGO genes, (2) differentially associated with distinct sets of synaptic genes.

### Biological functions derived from gene set enrichment analysis of Shank2-HT and Shank2-HM cortical transcripts

We next performed GSEA, wherein the whole set of transcripts is ranked by *p*-value and fold change with the goal of identifying biological functions that are influenced by many genes with moderate but coordinated transcriptional changes see footnote 4 (Mootha et al., 2003; Subramanian et al., 2005). This method avoids using an artificial and biased cutoff to identify a subset of transcripts for analysis, such as in DEG-based methods.

**FIGURE 4:**
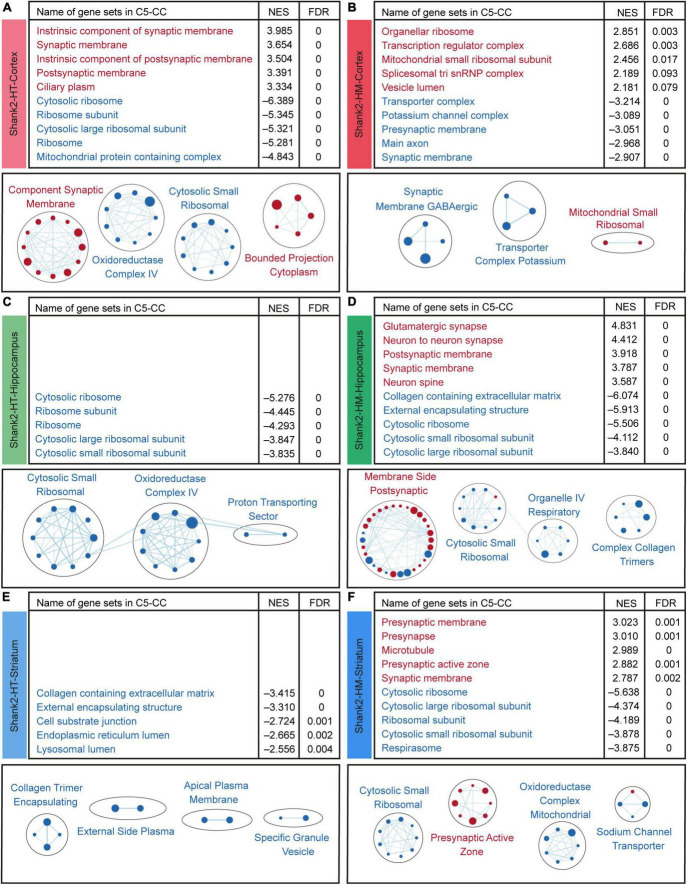
Biological functions derived from gene set enrichment analysis (GSEA) of Shank2-heterozygous (HT) and Shank2-homozygous (HM) cortical, hippocampal, and striatal transcripts. **(A–F)** GSEA results for Shank2-HT/HM cortical/hippocampal/striatal transcripts showing a list of the top five positively (red) and negatively (blue) enriched gene sets (top), and their integrated visualization generated using the Cytoscape App, EnrichmentMap (bottom). Only the results for C5-cellular component (CC) are shown here; those for C5-biological process (BP) and C5-molecular function (MF) are shown in Supplementary Figures 3–5. Note that only the top five gene sets are shown here (see Supplementary Table 3 for full results). In the EnrichmentMap results, each circle in a cluster indicates a significantly [false discovery rate (FDR) < 0.05] enriched gene set; the circle sizes and colors (red/blue) indicate the gene-set size and positive/negative enrichment based on NES scores, respectively [*n* = 5 mice (Shank2-HT/HM cortex, hippocampus, and striatum)].

Our GSEA results indicated that the Shank2-HT cortical transcripts are positively enriched for gene sets associated with neuronal synapses, as indicated by the top five gene sets enriched among those in the cellular component (CC) domain of the C5 gene sets (currently, ∼15,000 gene sets) (Figure 4A, top and Supplementary Table 3). This synaptic upregulation was further supported when we clustered the enriched gene sets using EnrichmentMap Cytoscape App (Figure 4A, bottom), a program that visualizes the functional clustering of the enriched gene sets (Merico et al., 2010; Isserlin et al., 2014). The Shank2-HT cortical transcripts were negatively enriched for gene sets associated with ribosomal and mitochondrial functions, as supported by the top five enriched gene sets and gene-set clustering by EnrichmentMap Cytoscape App (Figure 4A).

The Shank2-HM cortical transcripts were positively enriched for gene sets associated with ribosomes and mitochondria (Figure 4B), which contrasts with the negative enrichments of Shank2-HT transcripts for ribosome/mitochondria functions. In addition, the Shank2-HM cortical transcripts were negatively enriched for gene sets associated with neuronal synapses (Figure 4B). GSEA against the gene sets in the biological process (BP) and molecular function (MF) domains of the C5 database yielded partly similar results for Shank2-HT/HM cortical transcripts; negative enrichments of Shank2-HT cortical transcripts for ribosome/mitochondria-related gene sets in the BP domain, and negative enrichments of Shank2-HM cortical transcripts for potassium channel-related gene sets in the MF domain (Supplementary Figure 3).

These results suggest that Shank2-HT and Shank2-HM cortical transcripts show gene dosage-dependent distinct transcriptomic patterns that are associated with synaptic and ribosomal/mitochondrial functions and are largely opposite to each other.

### Biological functions derived from GSEA of Shank2-HT and Shank2-HM hippocampal transcripts

Surprisingly, our GSEA results for the Shank2-HT hippocampal transcripts did not identify any significant positive enrichment, as indicated by the top five enriched gene sets and EnrichmentMap Cytoscape results (Figure 4C and Supplementary Table 3). However, the negative enrichments of Shank2-HT hippocampal transcripts were significant for gene sets associated with ribosomes and mitochondria, as supported by the top five enriched gene sets and EnrichmentMap Cytoscape results (Figure 4C).

The Shank2-HM hippocampal transcripts were positively enriched for gene sets associated with neuronal synapses and negatively enriched for gene sets associated with the neuronal extracellular matrix (ECM), ribosomes, and mitochondria (Figure 4D). GSEA against the gene sets in the BP and MF domains of the C5 database yielded partly similar results for Shank2-HT/HM hippocampal transcripts; negative enrichments of Shank2-HT hippocampal transcripts for ribosome/mitochondria-related gene sets in the BP domain, and positive enrichments of the Shank2-HM hippocampal transcripts for synapse-related gene sets in the BP domain (Supplementary Figure 4).

These results suggest that Shank2-HT and Shank2-HM hippocampal transcripts show transcriptomic changes that are different from each other and also distinct from those observed in the cortex. For example, the synaptic gene upregulation observed in the Shank2-HT cortex was absent from the Shank2-HT hippocampus but present in the Shank2-HM hippocampus.

### Biological functions derived from GSEA analysis of Shank2-HT and Shank2-HM striatal transcripts

The GSEA results for the Shank2-HT striatal transcripts did not indicate any significant positive enrichment based on the top five enriched gene sets and EnrichmentMap Cytoscape results (Figure 4E and Supplementary Table 3). This was similar to the results obtained for Shank2-HT hippocampal transcripts. However, the Shank2-HT striatal transcripts were negatively and modestly enriched for gene sets associated with the ECM (Figure 4E).

The Shank2-HM striatal transcripts were positively enriched for gene sets associated with presynaptic genes (Figure 4F). In addition, the Shank2-HM striatal transcripts were negatively enriched for gene sets associated with neuronal ribosomes and mitochondria (Figure 4F). GSEA against the gene sets in the BP and MF domains of the C5 database yielded partly similar results for Shank2-HT/HM striatal transcripts; positive enrichments of Shank2-HM striatal transcripts for synapse-related gene sets and negative enrichments of the Shank2-HM striatal transcripts for ribosome/mitochondria-related gene sets in the BP domain (Supplementary Figure 5).

These results suggest that Shank2-HT and Shank2-HM striatal transcripts show transcriptomic changes that are distinct from each other and those observed in the cortex, but similar to those observed in the hippocampus. Notably, the enrichment patterns of the Shank2-HM striatal transcripts most closely resemble those of Shank2-HM hippocampal transcripts and Shank2-HT (not Shank2-HM) cortical transcripts.

### ASD-related patterns in Shank2-HT and Shank2-HM transcripts

Previous reports described transcriptomic changes associated with ASD (Garbett et al., 2008; Voineagu et al., 2011; Gupta et al., 2014; Parikshak et al., 2016; Velmeshev et al., 2019) and identified ASD-related gene sets that are up- or downregulated in ASD. These include DEG Up Voineagu, Co-Exp Up M16 Voineagu, DEG Down Voineagu, and Co-Exp Down M12 Voineagu (from cortical samples of individuals ranging in age from 2 to 560 years) (Voineagu et al., 2011; Werling et al., 2016). The genes in these gene sets are summarized in Supplementary Table 4.

Human genetic studies on ASD have also yielded various ASD-risk gene sets, including SFARI genes (Abrahams et al., 2013),^6^ FMRP targets (Darnell et al., 2011; Werling et al., 2016), DeNovoMissense (protein-disrupting or missense rare *de novo* variants) (Iossifov et al., 2014; Werling et al., 2016), DeNovoVariants (protein-disrupting rare *de novo* variants) (Iossifov et al., 2014; Werling et al., 2016), and AutismKB (Autism KnowledgeBase) (Xu et al., 2012; Yang et al., 2018; Supplementary Table 4). These genes are thought to be downregulated in ASD through mutations, including missense, nonsense, splice-site, frame-shift, and deletion mutations.

**FIGURE 5:**
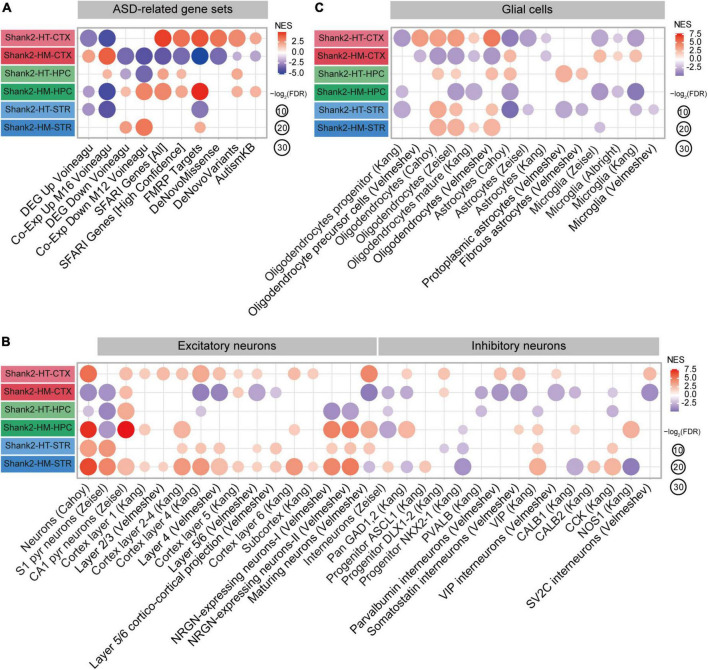
Autism spectrum disorder (ASD)-related patterns revealed by gene set enrichment analysis (GSEA) of Shank2-heterozygous (HT) and Shank2-homozygous (HM) cortical/hippocampal/striatal transcripts. **(A)** GSEA results for Shank2-HT/HM cortical/hippocampal/striatal transcripts, as shown by enrichment patterns for ASD-related gene sets that are upregulated in ASD (DEG Up Voineagu and Co-Exp Up M16 Voineagu) and downregulated in ASD (DEG Down Voineagu and Co-Exp Down M12 Voineagu) and ASD-risk gene sets [SFARI genes (all), SFARI genes (high confidence), FMRP targets, *DeNovo* Missense, *DeNovo* Variants, and AutismKB; *n* = 5 mice (Shank2-HT/HM cortex, hippocampus, and striatum)]. **(B)** GSEA results for Shank2-HT/HM cortical/hippocampal/striatal transcripts, as shown by enrichment patterns for cell-type-specific gene sets (glutamate and GABA neurons; *n* = 5 mice for Shank2-HT/HM cortex/hippocampus/striatum). **(C)** GSEA results for Shank2-HT/HM cortical/hippocampal/striatal transcripts, as shown by enrichment patterns for cell-type-specific gene sets (glial cells) [*n* = 5 mice (Shank2-HT/HM cortex, hippocampus, and striatum)].

Here, we used GSEA to test whether these ASD-related and ASD-risk gene sets were enriched in the Shank2-HT and Shank2-HM transcripts. In our GSEA for the cortex, the Shank2-HT transcripts were negatively enriched for ASD-related gene sets that are upregulated in ASD, such as DEG Up Voineagu and Co-Exp Up M16 Voineagu and positively enriched for ASD-risk gene sets, including SFARI genes (all and high confidence), FMRP targets, DeNovoMissense, DeNovoVariants, and AutismKB (Figure 5A and Supplementary Figure 6). These enrichment patterns are opposite to those observed in ASD (termed reverse-ASD patterns hereafter), and are likely to represent changes that arise to compensate for HT *Shank2* deletion.

In sharp contrast, Shank2-HM cortical transcripts were positively enriched for gene sets that are upregulated in ASD (DEG Up Voineagu and Co-Exp Up M16 Voineagu) and negatively enriched for gene sets that are downregulated in ASD (DEG Down Voineagu and Co-Exp Down M12 Voineagu) and for ASD-risk gene sets (SFARI genes, FMRP targets, DeNovoMissense, DeNovoVariants, and AutismKB) (Figure 5A). These patterns are similar to the transcriptomic changes occurring in ASD (termed ASD-like patterns hereafter).

The opposite changes in the enrichments of Shank2-HT and Shank2-HM cortical transcripts in two select gene sets [SFARI genes (all) and FMRP targets] were mediated by ∼50% of the genes in the two gene sets and were further supported by the small correlations of FC for co-up/down regulations (Supplementary Figure 7). The opposite changes in the SFARI genes, however, were weaker than those in FMRP target genes, as supported by the greater correlation coefficient (*r*^2^ = 0.1899 vs. 0.0425) involving frequent co-up/down regulations of more strongly changed transcripts. Collectively, these results indicate that HT and HM *Shank2* deletions in the cortex lead to largely opposite ASD-related/risk transcriptomic changes.

In the hippocampus, Shank2-HT transcripts showed both ASD-like and reverse-ASD patterns: They were positively enriched for the Co-Exp Up M16 Voineagu gene set, negatively enriched for the DEG Down Voineagu and Co-Exp Down M12 Voineagu gene sets (ASD-like patterns), and positively enriched for some of the ASD-risk gene sets (reverse-ASD patterns) (Figure 5A). In contrast, Shank2-HM hippocampal transcripts showed strong reverse-ASD patterns: They were negatively enriched for DEG Up Voineagu and Co-Exp Up M16 Voineagu and positively enriched for DEG Down Voineagu, Co-Exp Down M12 Voineagu, and all of the ASD-risk gene sets. Therefore, the hippocampal Shank2-HT and Shank2-HM patterns were distinct and the Shank2-HM hippocampal pattern (reverse-ASD) was similar to the Shank2-HT cortical pattern but largely opposite the Shank2-HM cortical pattern.

In the striatum, Shank2-HT and Shank2-HM transcripts showed patterns similar to those observed in the hippocampus, in that the Shank2-HT striatal transcripts showed a mixed pattern and the Shank2-HM striatal transcripts showed a reverse-ASD pattern. However, the overall extents of these changes were weaker than those observed in the hippocampus (Figure 5A). These results collectively suggest that the hippocampal and striatal transcriptomic patterns are more similar to each other than they are to the cortical patterns in *Shank2*-mutant mice.

### Cell-type-specific patterns in Shank2-HT and Shank2-HM transcripts

Cell-type-specific transcriptomic changes have been observed in ASD, including downregulation of neuron- and oligodendrocyte-related genes and upregulation of astrocyte- and microglia-related genes (Voineagu et al., 2011; Werling et al., 2016). We thus tested Shank2-HT and Shank2-HM transcripts for the cell-type-specific gene sets reported in previous studies (Albright and Gonzalez-Scarano, 2004; Cahoy et al., 2008; Kang et al., 2011; Zeisel et al., 2015; Werling et al., 2016; Velmeshev et al., 2019, 2020; Supplementary Table 4).

In the cortex, Shank2-HT transcripts were positively enriched for excitatory and inhibitory neuron-related gene sets as well as oligodendrocyte-related gene sets, as shown by enrichment patterns for single-cell-type gene sets (Figures 5B,C). In addition, Shank2-HT transcripts were negatively enriched for astrocyte- and microglia-related gene sets. These patterns were largely opposite those observed in ASD (reverse-ASD), and are in line with the abovementioned reverse-ASD patterns observed in our GSEA for ASD-related/risk gene sets (Figure 5A). In contrast, Shank2-HM cortical transcripts were overall negatively enriched for neuron (excitatory and inhibitory)- and oligodendrocyte-related gene sets and positively enriched for astrocyte- and microglia-related gene sets (Figures 5B,C), yielding a generally ASD-like pattern. These results from cell-type-specific gene set analyses suggest that an increase in the *Shank2* deletional dosage converts a reverse-ASD pattern to an ASD-like pattern, as seen for our GSEA results on ASD-related/risk gene sets (Figure 5A).

In the hippocampus, Shank2-HT transcripts displayed a mixed pattern (both ASD-like and reverse-ASD), as supported by largely negative enrichments for neuron-related gene sets, positive enrichments for oligodendrocyte-related gene sets, and moderate positive enrichments for astrocyte/microglia-related gene sets (Figures 5B,C). Shank2-HM transcripts showed a reverse-ASD pattern, but it was still mixed, displaying largely positive enrichments for neuron-related gene sets, negative enrichments for oligodendrocyte-related gene sets, and negative enrichments for astrocyte/microglia-related gene sets (Figures 5B,C). Therefore, unlike our observation in the cortex, an increase in the *Shank2* deletional dosage intensified the reverse-ASD pattern in the hippocampus.

In the striatum, Shank2-HT transcripts showed a reverse-ASD pattern with largely positive enrichments for neuron/oligodendrocyte-related gene sets and negative enrichments for astrocyte/microglia-related gene sets (Figures 5B,C). Shank2-HM transcripts showed a similar reverse-ASD pattern with positive enrichments for neuron/oligodendrocyte-related gene sets and a moderately negative enrichment for microglia-related genes (Figures 5B,C). Therefore, HT and HM *Shank2* deletion in the striatum lead to similar reverse-ASD patterns.

These results collectively suggest that HT and HM *Shank2* deletions lead to distinct gene dosage effects on ASD-related/risk gene expressions in the cortex and hippocampus but not in the striatum; increased *Shank2* deletional dosage intensifies the ASD-like pattern in the cortex, weakens it in the hippocampus, and has no effect on it in the striatum.

## Discussion

In the current study, we investigated transcriptomic changes occurring in three different brain regions (prefrontal cortex, hippocampus, and striatum) of adult Shank2-HT/HM mice. The results indicate that there are brain region and gene dosage-differential transcriptomic changes associated with altered biological functions and ASD-related/risk gene expression patterns.

### Transcriptomic changes in *Shank2*-mutant mice

The results of our DEG and DAVID analyses indicated that the hippocampal and striatal Shank2-HM DEGs are differentially associated with synapse- and neuronal subregion/substructure-related functions (Figures 1, 2). In addition, our SynGO and PPI analyses indicated that hippocampal and striatal Shank2-HM DEGs belonging to SynGO genes are differentially enriched for pre- and postsynaptic functions and form differential PPI networks (Figure 3). For instance, the hippocampal SynGO-DEGs formed a PPI cluster of mainly upregulated synaptic genes (with the exception of ribosome-related genes), whereas the striatal SynGO-DEGs formed a cluster of synaptic genes with mixed up and downregulations. Although additional details remain to be determined, these results suggest that different *Shank2*-mutant brain regions (hippocampus and striatum) respond to *Shank2* deletion by distinct up/downregulations and *via* different PPI networks of synaptic genes.

The DEG results also indicate that there are more DEGs in Shank2-HM transcripts compared to Shank2-HT transcripts, regardless of the brain region examined (Figure 1). Our previous study showed that Shank2-HT mice display largely normal behaviors, whereas Shank2-HM mice show strong autistic-like behaviors (Won et al., 2012). We therefore speculate that the greater numbers of DEGs in these three brain regions of Shank2-HM mice relative to Shank2-HT mice may collectively contribute to the mechanistic deviations that underlie the observed abnormal behaviors. An alternative and not mutually exclusive explanation could be that the greater numbers of DEGs in Shank2-HM transcripts relative to Shank2-HT transcripts may reflect that stronger compensatory transcriptomic responses are induced by a stronger mutation.

Gene set enrichment analysis of the cortical Shank2-HT transcripts showed a reverse-ASD pattern associated with increased synapse-related gene expression (Figures 4, 5). In contrast, the Shank2-HM transcripts showed an ASD-like pattern associated with decreased synaptic gene expression. It is possible that the increased synaptic gene expression in Shank2-HT mice may be compensatory in nature and may underlie the largely normal behaviors seen in these mice, whereas the decreased synaptic gene expression in Shank2-HM mice associated with the ASD-like transcriptomic pattern may promote the behavioral abnormalities seen in these mice. In line with this hypothesis, Shank2-HM mice show impaired neuronal responses to social contexts in the medial prefrontal cortex (mPFC), which has been strongly implicated in ASD (Yizhar et al., 2011; Yan and Rein, 2021); this impairment involves decreased inhibition of target pyramidal neurons by parvalbumin-positive interneurons (Lee et al., 2021a).

When the current GSEA results for Shank2-HT and Shank2-HM cortical/prefrontal transcripts, covering wider areas of the prefrontal cortex including more caudal regions, are compared with the previous results for Shank2-HT and Shank2-HM mPFC transcripts (Lee et al., 2021b) for ASD-related/risk patterns, the reverse-ASD pattern of the current Shank2-HT (but not Shank2-HM showing ASD-like pattern) prefrontal transcripts are more similar to the previous results (reverse-ASD for both Shank2-HT and Shank2-HM mPFC transcripts). It might be possible that the mPFC region may be more resilient in inducing compensatory and reverse-ASD transcriptomic changes even in the presence of a strong HM *Shank2* deletion.

Gene set enrichment analysis of the hippocampal Shank2-HT transcripts revealed mixed ASD-like and reverse-ASD patterns associated with largely normal synapse-related gene expression levels. In contrast, the Shank2-HM transcripts showed a strong reverse-ASD pattern associated with increased synaptic and FMRP target gene expression levels, as well as increased excitatory neuronal gene expression and decreased glial (astrocytic and microglial) gene expression (Figures 4, 5). Previous studies on adult Shank2-HM mice revealed the presence of impaired hippocampal synaptic functions that have been causally associated with behavioral deficits (Won et al., 2012; Lee et al., 2015b) and early postnatal NMDAR hyperfunction (Chung et al., 2019; Yoo et al., 2021). In addition, reduced hippocampal inhibitory synaptic transmission has been causally associated with impaired spatial memory in *Shank2*-mutant mice that lack exons 6–7 (Lim et al., 2017). It is therefore possible that the strong reverse-ASD pattern observed in Shank2-HM hippocampal transcripts, which was absent from Shank2-HT hippocampal transcripts, may represent compensatory changes that occur in response to a stronger *Shank2* deletion and but fail to fully rescue the hippocampal synaptic deficits. This sharply contrasts with the abovementioned hypothesis that the reverse-ASD patterns seen in the Shank2-HT cortex may represent transcriptomic changes that successfully contribute to normalizing behavioral phenotypes.

In the striatum, the Shank2-HT transcripts also showed mixed ASD-like and reverse-ASD patterns associated with largely normal synaptic gene expression levels. The Shank2-HM transcripts, however, showed a strong reverse-ASD pattern associated with increased synaptic and excitatory neuronal (both excitatory and inhibitory) gene expression levels (Figures 4, 5). A notable difference between the Shank2-HM hippocampal and striatal transcripts is that the same reverse-ASD patterns seem to more strongly involve ASD-related/risk gene sets in the hippocampus but cell type-specific gene sets in the striatum, suggesting that there are distinct molecular/cellular and neuronal mechanisms.

In summary, our results indicate that *Shank2* deletion leads to brain region and gene dosage-differential transcriptomic changes associated with altered biological functions and ASD-related/risk gene expression patterns. These results provide unbiased clues on the mechanisms underlying the ASD-related phenotypes in *Shank2*-mutant mice and will be useful in designing future experiments using these mice and interpreting the results.

## Data availability statement

The datasets presented in this study can be found in online repositories. The raw RNA-Seq results are available as GSE200439 (*Shank2* brain regions) at GEO (Gene Expression Omnibus), NCBI (National Center for Biotechnology Information).

## Ethics statement

The animal study was reviewed and approved by the Committee of Animal Research at KAIST (KA2020-99).

## Author contributions

Y-EY, TY, and EK designed the experiments. Y-EY, TY, and HK performed the RNA-seq analyses. HK and EK wrote the manuscript. All authors contributed to the article and approved the submitted version.

## Footnotes

1
https://david.ncifcrf.gov/

2
https://www.syngoportal.org/

3
https://string-db.org/

4
http://software.broadinstitute.org/gsea

5
http://software.broadinstitute.org/gsea/msigdb

6
https://gene.sfari.org/

## Supplementary material

Supplementary Figure 1 Clustering of three brain regional transcripts from Shank2-heterozygous (HT)/homozygous (HM) mice by heatmap analysis. Transcript groups from the three brain regions (cortex, hippocampus, and striatum) of Shank2-wild-type (WT), Shank2-HT, and Shank2-HM mice were analyzed by a heatmap analysis [13 weeks; male; *n* = 5 mice (WT), 5 (HT), and 5 (HM)].

Supplementary Figure 2 Small correlations of fold changes (FC) between the transcript groups from two different brain regions. **(A–C)** Correlograms of the FC of the transcripts from two brain regions, in which one group contains transcripts that are differentially expressed genes (DEGs) in one brain region but not in another brain region, indicate small correlation coefficients, except for the transcripts that are DEGs in both brain regions [*n* = 5 mice wild-type (WT), 5 heterozygous (HT), and 5 homozygous (HM), Pearson test).

Supplementary Figure 3 Biological functions derived from gene set enrichment analysis (GSEA) of Shank2-heterozygous (HT) and Shank2-homozygous (HM) cortical transcripts using gene sets in the C5-biological process (BP) and C5-molecular function (MF) domains. **(A–D)** GSEA results for Shank2-HT/HM cortical transcripts showing a list of the top five positively (red) and negatively (blue) enriched gene sets (top), and their integrated visualization generated using the Cytoscape App, EnrichmentMap (bottom). Note that only the top five gene sets are shown here (see Supplementary Table 3 for full results). Circles in the EnrichmentMap results indicate significantly [false discovery rate (FDR) < 0.05] enriched individual gene sets, with circle sizes and colors (red/blue) indicating gene-set size and positive/negative enrichment based on normalized enrichment score (NES) scores, respectively [*n* = 5 mice (Shank2-HT/HM cortex)].

Supplementary Figure 4 Biological functions derived from gene set enrichment analysis (GSEA) of Shank2-heterozygous (HT) and Shank2-homozygous (HM) hippocampal transcripts using gene sets in the C5-biological process (BP) and C5-molecular function (MF) domains. **(A–D)** GSEA results for Shank2-HT/HM hippocampal transcripts showing a list of the top five positively (red) and negatively (blue) enriched gene sets (top), and their integrated visualization generated using the Cytoscape App, EnrichmentMap (bottom) [*n* = 5 mice (Shank2-HT/HM hippocampus)].

Supplementary Figure 5 Biological functions derived from gene set enrichment analysis (GSEA) of Shank2-heterozygous (HT) and Shank2-homozygous (HM) striatal transcripts using gene sets in the C5-biological process (BP) and C5-molecular function (MF) domains. **(A–D)** GSEA results for Shank2-HT/HM striatal transcripts showing a list of the top five positively (red) and negatively (blue) enriched gene sets (top), and their integrated visualization generated using the Cytoscape App, EnrichmentMap (bottom) [*n* = 5 mice (Shank2-HT/HM striatum)].

Supplementary Figure 6 Autism spectrum disorder (ASD)-related patterns revealed by gene set enrichment analysis (GSEA) of Shank2-heterozygous (HT) and Shank2-homozygous (HM) cortical/hippocampal/striatal transcripts. **(A–C)** GSEA results shown in Figure 5 are shown here again with the indication of color-coded NES scores for insignificantly enriched gene sets in square dots, together with circular dots (significant enrichments). Note that the normalized enrichment score (NES) scores in the insignificant enrichments are smaller than those for significant enrichments [*n* = 5 mice (Shank2-HT/HM cortex, hippocampus, and striatum)].

Supplementary Figure 7 Analysis of individual gene expression patterns for opposite enrichments of Shank2-heterozygous (HT) and Shank2-homozygous (HM) cortical transcripts for two select autism spectrum disorder (ASD)-risk gene sets. **(A,B)** The opposite enrichments of Shank2-HT and Shank2-HM cortical transcripts for two select ASD-risk gene sets [SFARI genes (all) and FMRP targets] were mediated by a large portion (∼50%) of the genes in the gene sets **(A)** and were further supported by the small correlations of the fold changes for co-up/down regulations **(B)** [*n* = 5 mice (Shank2-HT/HM cortex), Pearson test].
