# Supplementary figures and images for: Brain region and gene dosage-differential transcriptomic changes in Shank2-mutant mice
Source: Front Mol Neurosci. 2022 Oct 13;15:977305. doi: 10.3389/fnmol.2022.977305 (PMC9612946; doi:10.3389/fnmol.2022.977305)

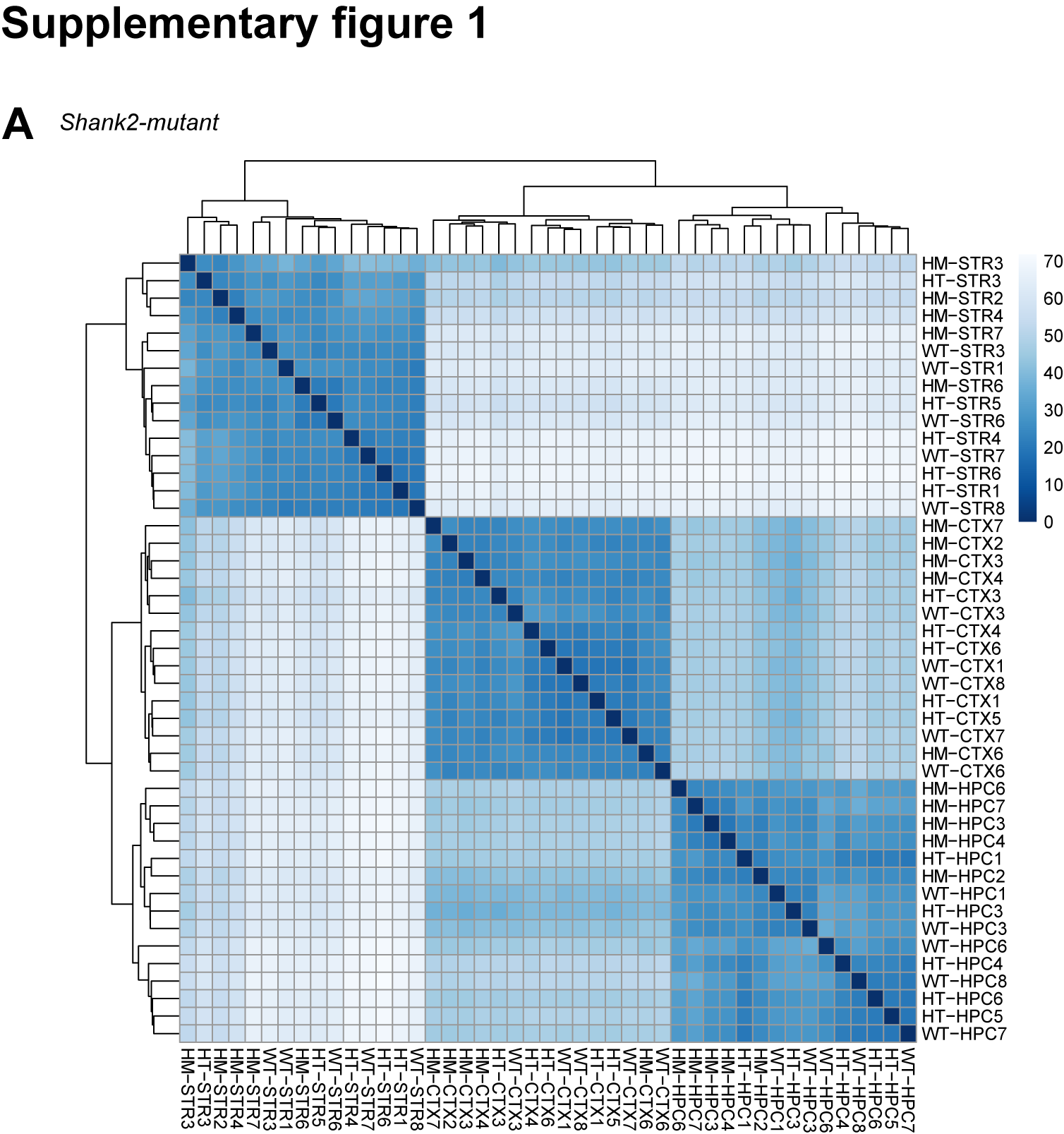

Supplement: Supplementary Figure 1 — Clustering of three brain regional transcripts from Shank2-heterozygous (HT)/homozygous (HM) mice by heatmap analysis. Transcript groups from the three brain regions (cortex, hippocampus, and striatum) of Shank2-wild-type (WT), Shank2-HT, and Shank2-HM mice were analyzed by a heatmap analysis [13 weeks; male; n = 5 mice (WT), 5 (HT), and 5 (HM)]. [file Image_1.TIF]

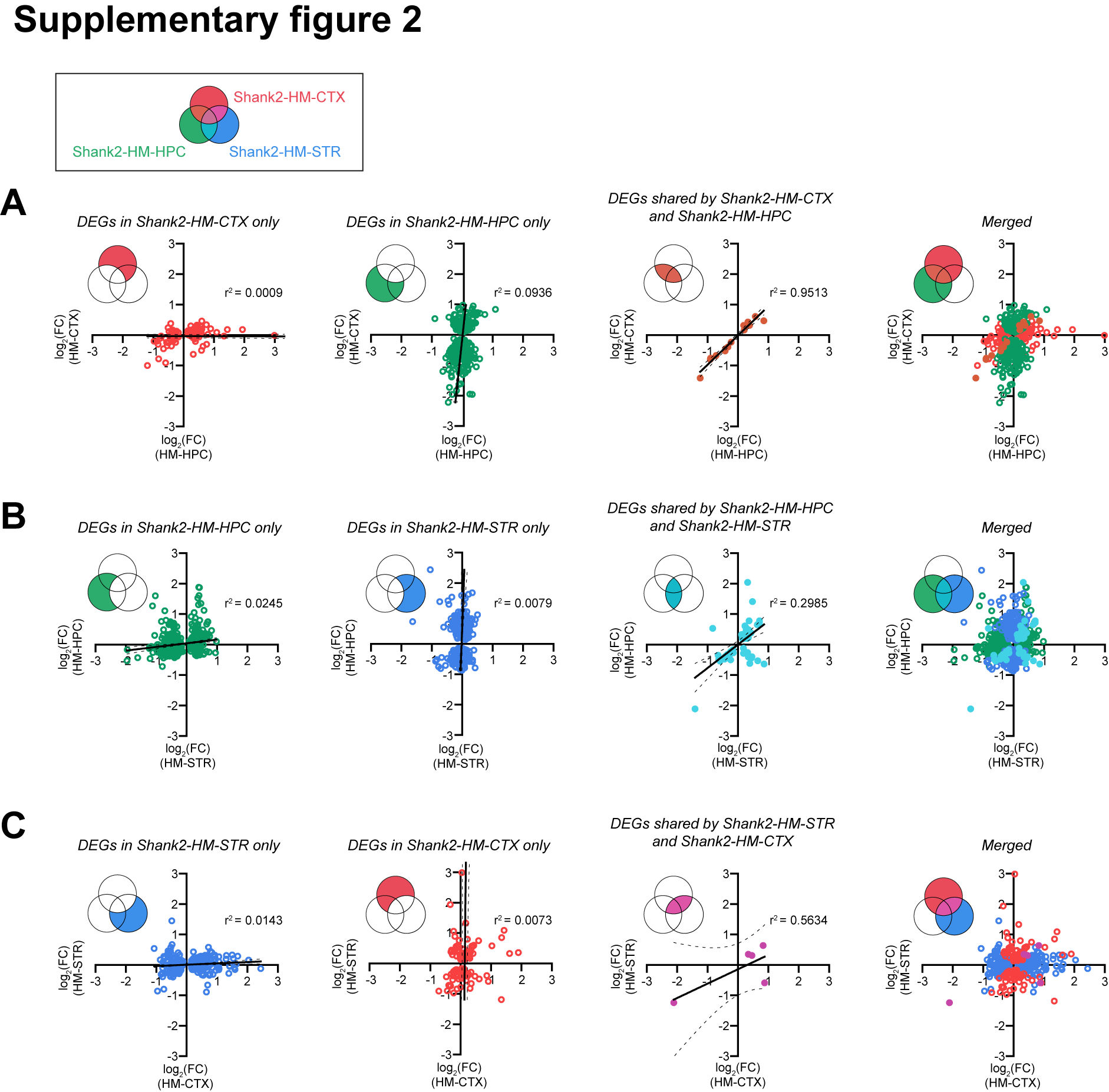

Supplement: Supplementary Figure 2 — Small correlations of fold changes (FC) between the transcript groups from two different brain regions. (A–C) Correlograms of the FC of the transcripts from two brain regions, in which one group contains transcripts that are differentially expressed genes (DEGs) in one brain region but not in another brain region, indicate small correlation coefficients, except for the transcripts that are DEGs in both brain regions [n = 5 mice wild-type (WT), 5 heterozygous (HT), and 5 homozygous (HM), Pearson test). [file Image_2.TIF]

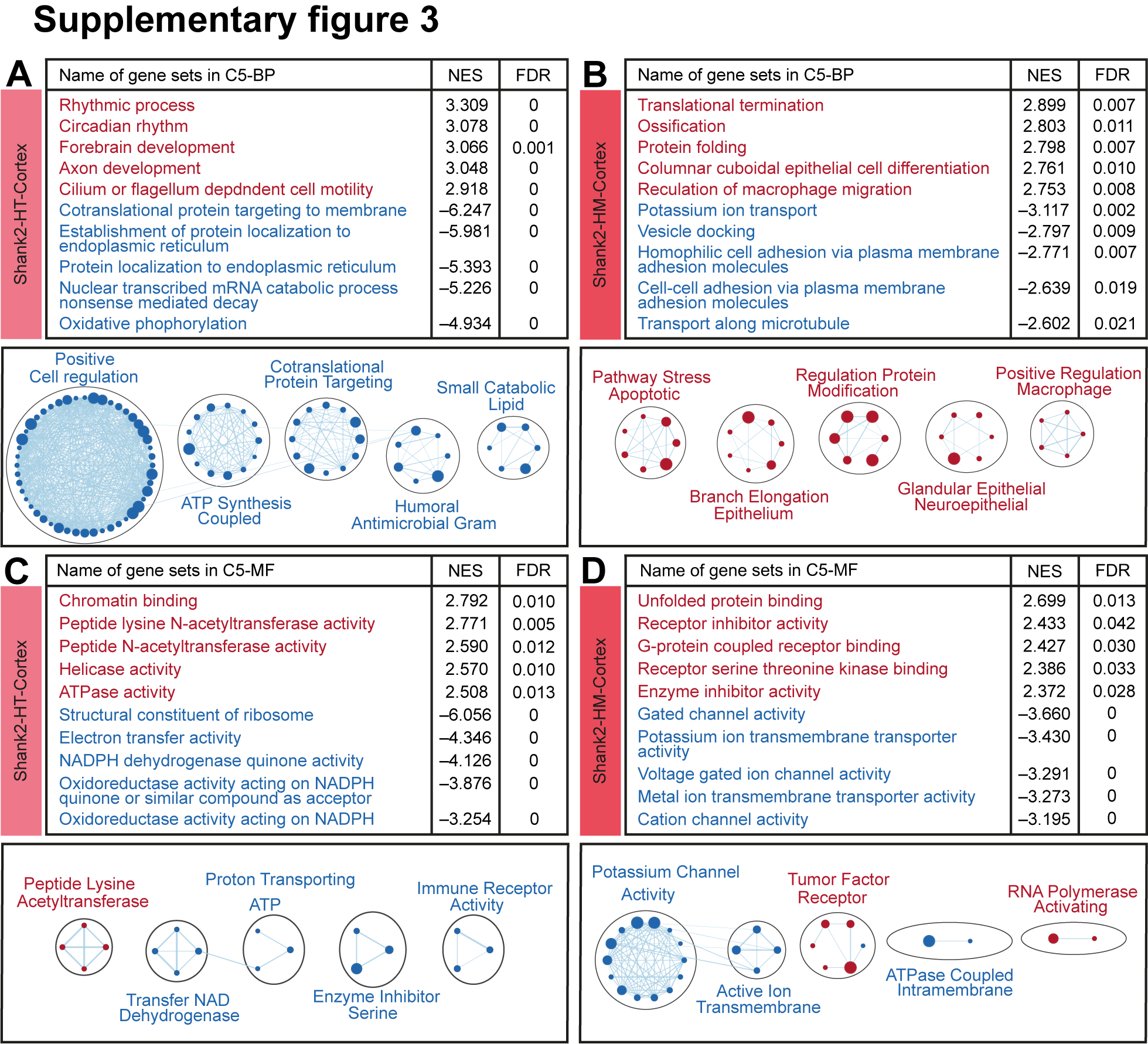

Supplement: Supplementary Figure 3 — Biological functions derived from gene set enrichment analysis (GSEA) of Shank2-heterozygous (HT) and Shank2-homozygous (HM) cortical transcripts using gene sets in the C5-biological process (BP) and C5-molecular function (MF) domains. (A–D) GSEA results for Shank2-HT/HM cortical transcripts showing a list of the top five positively (red) and negatively (blue) enriched gene sets (top), and their integrated visualization generated using the Cytoscape App, EnrichmentMap (bottom). Note that only the top five gene sets are shown here (see Supplementary Table 3 for full results). Circles in the EnrichmentMap results indicate significantly [false discovery rate (FDR) < 0.05] enriched individual gene sets, with circle sizes and colors (red/blue) indicating gene-set size and positive/negative enrichment based on normalized enrichment score (NES) scores, respectively [n = 5 mice (Shank2-HT/HM cortex)]. [file Image_3.TIF]

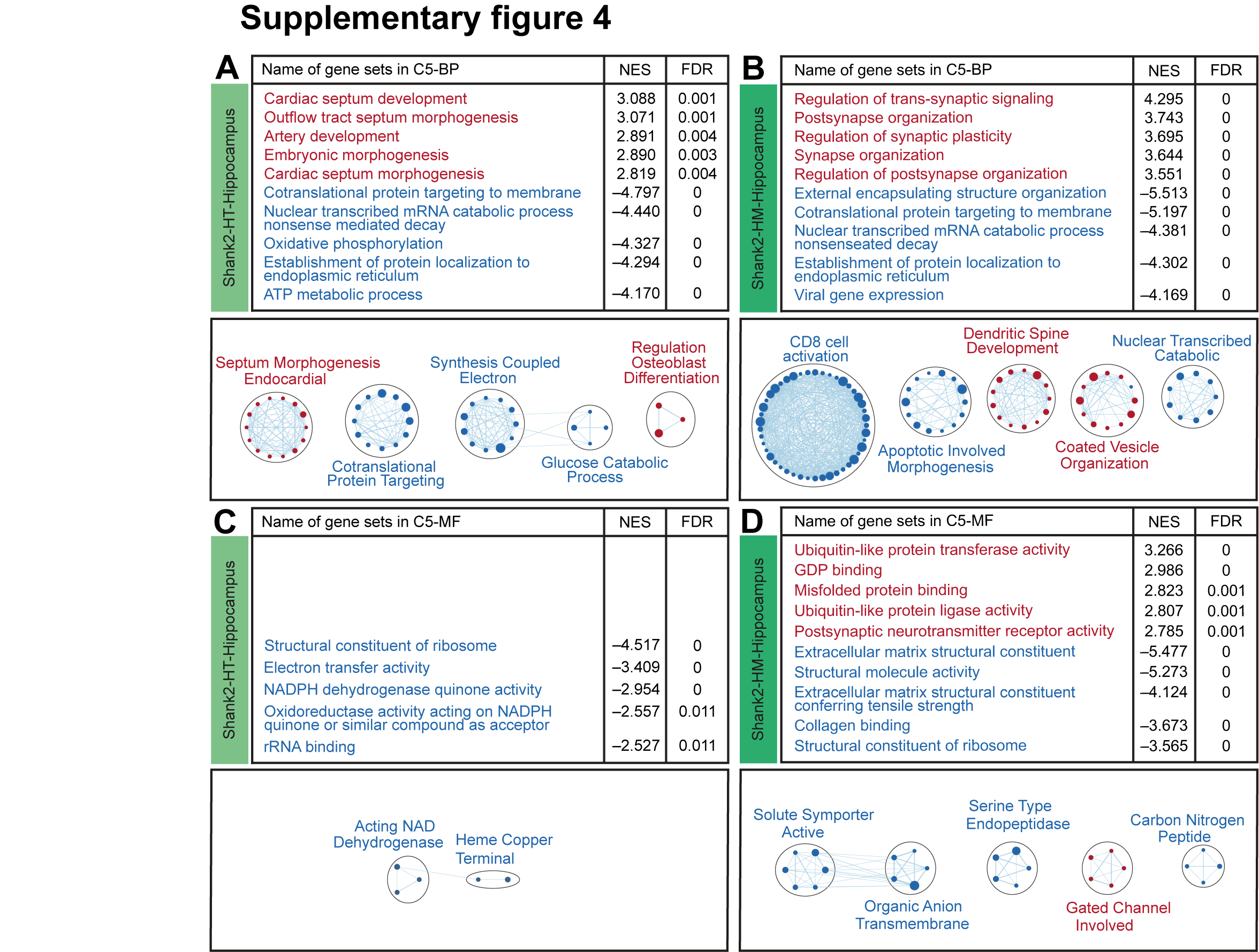

Supplement: Supplementary Figure 4 — Biological functions derived from gene set enrichment analysis (GSEA) of Shank2-heterozygous (HT) and Shank2-homozygous (HM) hippocampal transcripts using gene sets in the C5-biological process (BP) and C5-molecular function (MF) domains. (A–D) GSEA results for Shank2-HT/HM hippocampal transcripts showing a list of the top five positively (red) and negatively (blue) enriched gene sets (top), and their integrated visualization generated using the Cytoscape App, EnrichmentMap (bottom) [n = 5 mice (Shank2-HT/HM hippocampus)]. [file Image_4.TIF]

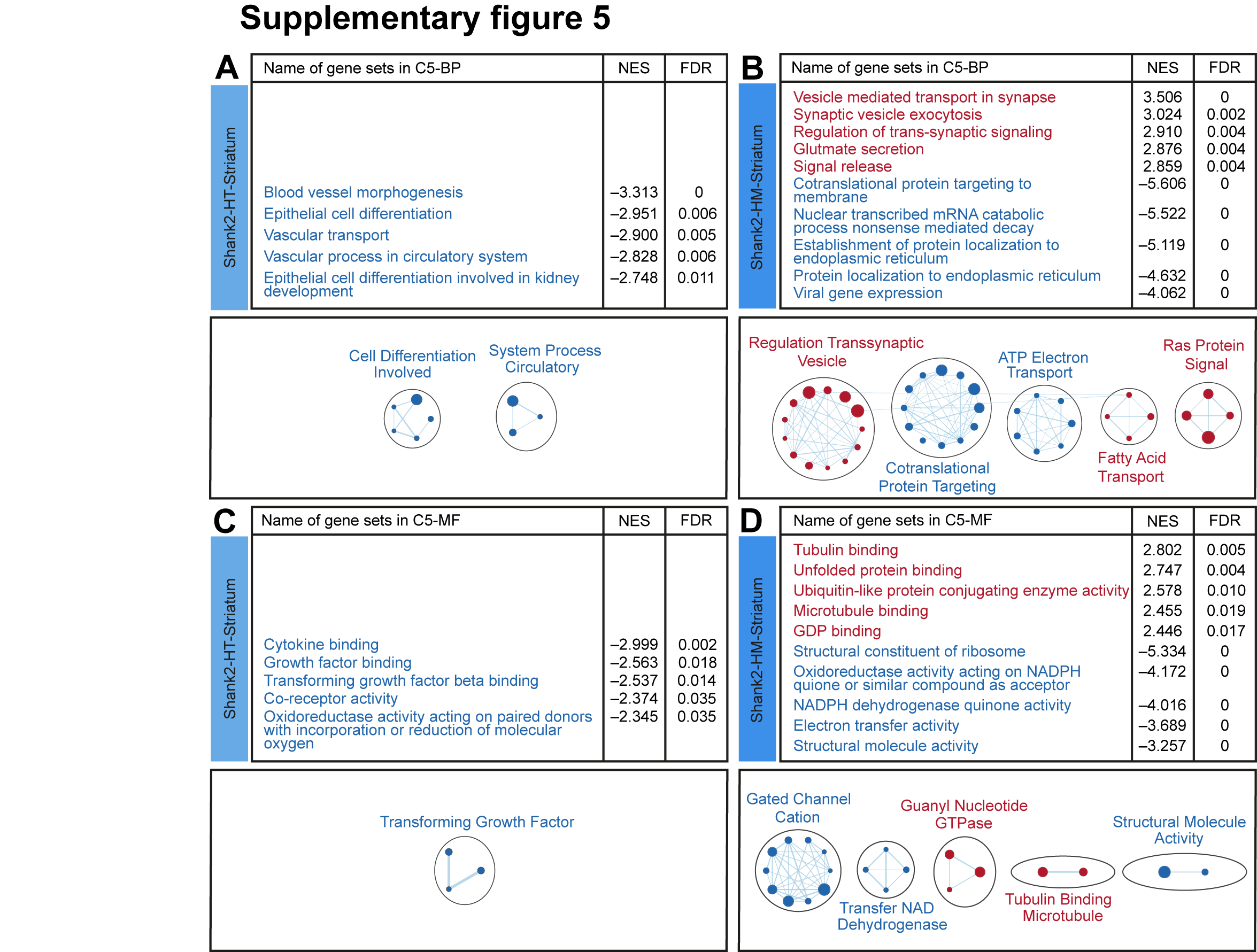

Supplement: Supplementary Figure 5 — Biological functions derived from gene set enrichment analysis (GSEA) of Shank2-heterozygous (HT) and Shank2-homozygous (HM) striatal transcripts using gene sets in the C5-biological process (BP) and C5-molecular function (MF) domains. (A–D) GSEA results for Shank2-HT/HM striatal transcripts showing a list of the top five positively (red) and negatively (blue) enriched gene sets (top), and their integrated visualization generated using the Cytoscape App, EnrichmentMap (bottom) [n = 5 mice (Shank2-HT/HM striatum)]. [file Image_5.TIF]

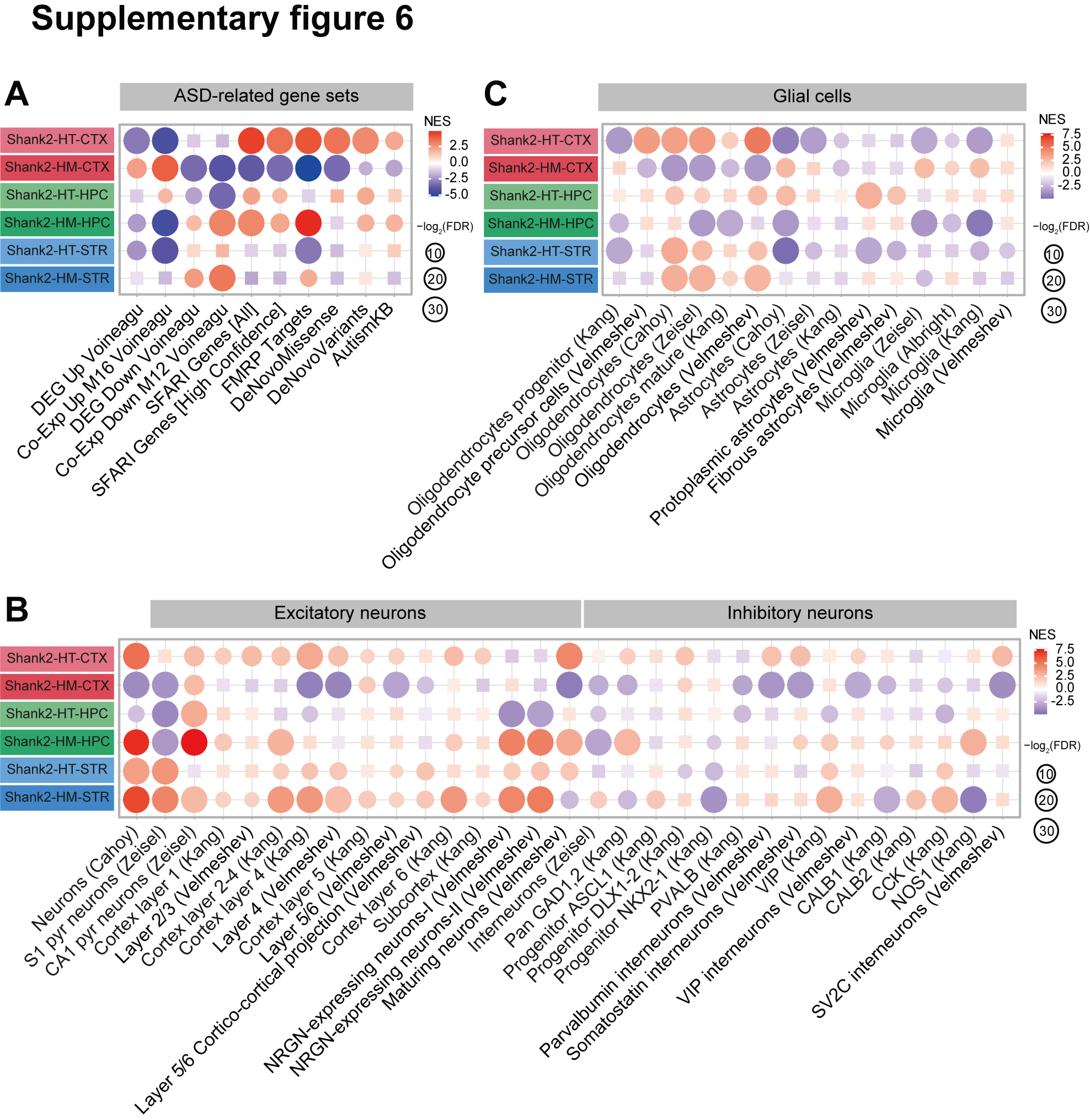

Supplement: Supplementary Figure 6 — Autism spectrum disorder (ASD)-related patterns revealed by gene set enrichment analysis (GSEA) of Shank2-heterozygous (HT) and Shank2-homozygous (HM) cortical/hippocampal/striatal transcripts. (A–C) GSEA results shown in Figure 5 are shown here again with the indication of color-coded NES scores for insignificantly enriched gene sets in square dots, together with circular dots (significant enrichments). Note that the normalized enrichment score (NES) scores in the insignificant enrichments are smaller than those for significant enrichments [n = 5 mice (Shank2-HT/HM cortex, hippocampus, and striatum)]. [file Image_6.TIF]

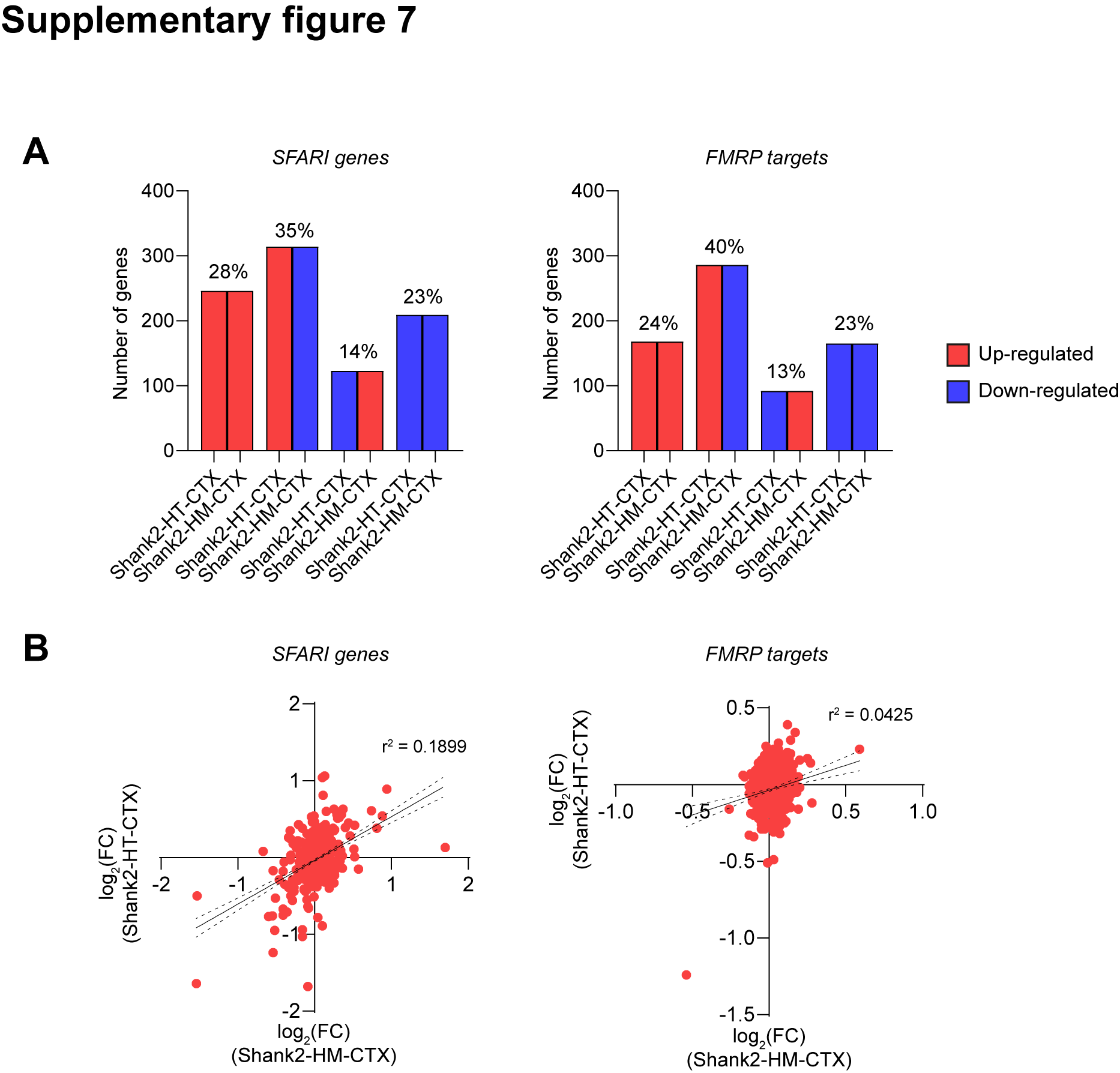

Supplement: Supplementary Figure 7 — Analysis of individual gene expression patterns for opposite enrichments of Shank2-heterozygous (HT) and Shank2-homozygous (HM) cortical transcripts for two select autism spectrum disorder (ASD)-risk gene sets. (A,B) The opposite enrichments of Shank2-HT and Shank2-HM cortical transcripts for two select ASD-risk gene sets [SFARI genes (all) and FMRP targets] were mediated by a large portion (∼50%) of the genes in the gene sets (A) and were further supported by the small correlations of the fold changes for co-up/down regulations (B) [n = 5 mice (Shank2-HT/HM cortex), Pearson test]. [file Image_7.TIF]
